# Fractionation and Hydrolyzation of Avocado Peel Extract: Improvement of Antibacterial Activity

**DOI:** 10.3390/antibiotics10010023

**Published:** 2020-12-30

**Authors:** Igor Trujillo-Mayol, Nidia Casas-Forero, Edgar Pastene-Navarrete, Fabiana Lima Silva, Julio Alarcón-Enos

**Affiliations:** 1 Food Engineering Department, Health and Food Science Faculty, Universidad del Bío-Bío, Av. Andrés Bello 720, PO Box 447, Chillan 3780000, Chile; igor.trujillo1601@alumnos.ubiobio.cl (I.T.-M.); nidia.casas1701@alumnos.ubiobio.cl (N.C.-F.); 2 Laboratory of Synthesis and Biotransformation of Natural Products, Faculty of Science, Universidad del Bío-Bío, Av. Andrés Bello 720, PO Box 447, Chillan 3780000, Chile; epastene@ubiobio.cl; 3 Institute of Health Sciences, Universidade Paulista, São Paulo 13565-905, Brazil; fabiana.silva@docente.unip.br; 4 Faculty of Basic Sciences, Universidad del Bío-Bío Campus Fernando May, Av. Andrés Bello 720, Chillan 3800708, Chile

**Keywords:** antioxidants, biofilm inhibition, phenolic compounds, avocado peel, microwave

## Abstract

Avocado Hass (*Persea americana* Mill) peel extract (APE) has the potential as a natural ingredient to substitute for chemical preservatives. The objectives of this study were to assess the phytochemical composition by high-performance liquid chromatography–quadrupole time-of-flight mass/mass spectrometry (HPLC-qTOF-MS/MS), total phenolic content (TPC), proanthocyanidin (PAC) content, and antioxidant activity of the APE, the organic fraction (OF), the aqueous fraction (AF), and the acid-microwave hydrolyzed APE (HAPE), on the antibacterial activity (ABA). The results indicated that APE and OF contained (*p* ˂ 0.05) a higher phenolic composition and antioxidant activity than AF and HAPE. The ABA specified that *Pseudomonas aeruginosa* and *Bacillus cereus* were inhibited by all the extracts (minimal inhibitory concentration—MIC ≥ 500 µg/mL), *Staphylococcus aureus* was only significantly inhibited by APE (≥750 µg/mL), the same MIC was observed for the OF on *Salmonella* spp. and *Listeria monocytogenes*. The HAPE increased the inhibitory efficiency up to 25% on *Escherichia coli* and *Salmonella* spp. (MIC ≥ 750 µg/mL), and 83.34% on *L. monocytogenes* (MIC ≥ 125 µg/mL) compared to APE (MIC ≥ 750 µg/mL). Also, HAPE inhibited the biofilm formation at the lowest concentration (125 µg/mL); meanwhile, the biofilm disruption showed to be concentration-time-dependent (*p* ˃ 0.05) compared to amoxicillin. In conclusion, the fractionation and hydrolyzation of APE improved the ABA; thus, those strategies are useful to design new antimicrobial compounds.

## 1. Introduction

The fight against food oxidation and dangerous Gram-negative and positive bacteria is still a challenge. Consequently, multiple efforts are focused on the inhibition of bacteria in planktonic and sessile states. Such bacterial forms are responsible for multiple outbreaks with several negative repercussions on human health and the economy [1]. Hence, the concerns about foodborne illnesses and the possible adverse effect of synthetic preservatives added to food opened the door to incorporating plant extracts high in phenolic compounds (PCs) obtained from fruit by-products such as avocado peel [2,3]. These biomolecules are commonly categorized into two groups: non-flavonoids (phenolic acids, stilbenes, and lignans) and flavonoids (flavones, flavanols, flavanones, anthocyanidins, isoflavones, flavonols, and proanthocyanidins) [2,4,5]. The latter group has particularly attracted much attention due to the high antibacterial activity (ABA) [6].

The phenolic content and composition are responsible for the antioxidant and ABA in plant extracts. The mode of action against food poisoning bacteria is tightly related to the plant extract’s phenolic composition and the target bacteria’s physiology, especially the membrane [7,8,9]. The PCs’ hydroxylation patterns are thought to be a key factor for bacteria toxicity [10]. In flavonoids, the double bond between the C_2_ and C_3_, the catechol group of B ring, the 5/7 of OH position on the A ring, the 3-OH group in the C ring, and in general, the amount of hydroxyl groups play a pivotal role in the antioxidant activity and bacterial inhibition [11]. Moreover, for proanthocyanidins (PACs), the polymerization degree rules the biological activity [12]. Regarding phenolic acids (PA), besides the number of hydroxyls directly linked to the aromatic ring, the ABA is given by the length of the carbon side chain [13]. Even though in nature, PCs, especially flavonoids, are bound to sugar moieties and PA, the aglycones display higher antioxidant and ABA, though not generalized [4,11,14]. As a whole, the amount and location of OH groups, the double bonds, the polymerization degree, the chain length, and the lack of acylation increase the PCs’ hydrophobicity; thus, the ABA increases as well, although, the antioxidant activity can be affected.

Raw extracts from different by-products have exhibited minimal inhibitory concentration (MIC) against food pathogen bacteria from 25 to 5000 µg/mL [15]. Nevertheless, samples with an MIC around 500 µg/mL are considered to own considerable ABA, and above 1000 µg/mL are not clinically relevant [16,17]. To increase the biological activity of PCs, very few approaches have been performed. To release esterified and glycosylated phenolics in plant extracts, acidic hydrolysis catalyzed by temperature, followed by fractionation with less polar solvents, has been performed to modify the antioxidant activity of plant extract [14,18,19,20]. In addition, it was observed that weak polar phenolic fractions displayed higher antimicrobial activity [16,19,20,21,22]. For instance, the extraction of PAC with ethyl acetate from laurel wood increased the anti-*Listeria monocytogenes* and other foodborne pathogen activity; the biofilms were inhibited and disrupted, as well [23]. Related studies were reported elsewhere against Gram (-) and (+) [6]. Similarly, the purification of PAC from avocado peel enhanced the inhibition of *Helicobacter pillory* [24]. However, studies related to the effect of hydrolysates extracts on the antibacterial activity were not found.

The avocado peel extract (APE) is a valued source of PCs that can inhibit oxidative reactions and microbial growth. Selectivity and moderate ABA characterize the APE, though the antioxidant activity is high. Our previous work demonstrated the effect of the eco-friendly extraction methods on the biological activity (antioxidant and antimicrobial) of hydroethanolic APE [3]. The results indicated considerable inhibition of *Bacillus cereus (B. cereus)*, moderate (MIC ≤ 1000 µg/mL) activity against *Escherichia coli (E. coli)*, *Staphylococcus aureus (S. aureus)*, *Salmonella* spp., *Listeria monocytogenes (L. monocytogenes)*. Concerns were raised due to the limited ABA of APE, especially against the last bacteria, due to the multiple problems it brings to the food industry and human health. Bearing in mind the above discussed, it was suspected that the hydrolyzation and the fractionation with weak polar solvent would increase the APE’s biological activity.

Accordingly, the objectives of the present study were to fractionate the APE with a weak polar solvent, then to perform acidic hydrolysis catalyzed by microwaves, and to re-extract with ethyl acetate the APE hydrolysate. This is followed by characterizing the phytochemical composition of the APE, the organic fraction (OF), the aqueous fraction (AF), and the organic fraction of the hydrolyzed APE (HAPE) by high-performance liquid chromatography–quadrupole time-of-flight mass/mass spectrometry (HPLC-qTOF-MS/MS), to address molecular differences of each fraction, as well as to determine the total phenolic content (TPC), proanthocyanidin (PAC) content, the antioxidant activity measured in terms of the 2,2-Diphenyl-1-picrylhydrazyl (DPPH) scavenging capacity, ferric reducing power (FRAP), and oxygen radical absorbance capacity (ORAC). Finally, it is necessary to assess the ABA on the common Gram (−) and (+) food pathogenic bacteria and the inhibition and disruption capacity against the *L. monocytogenes* biofilms. This study provides valuable information about the biological improvements in phenolic extracts, especially when subjected to thermic treatments and fractionation.

## 2. Results

### 2.1. Phytochemical and Antioxidant Characterization

#### 2.1.1. HPLC-ESI-qTOF-MS/MS Characterization

**Figure 1:**
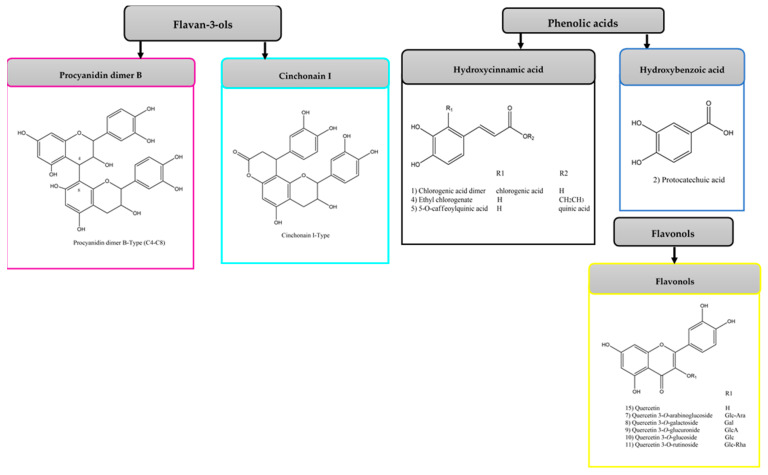
Molecular structures of the main groups of compounds characterized in avocado peel extract (APE), organic fraction (OF), aqueous fraction (AF), and the organic fraction of acid-microwave hydrolyzed avocado peel extract (HAPE).

APE obtained by the ultrasound-microwave combined method and its fractions, AF, OF, and organic fraction of the acid-microwave hydrolyzed APE (HAPE) were analyzed by high-performance liquid chromatography–quadrupole time-of-flight mass/mass spectrometry (HPLC-ESI-qTOF-MS/MS) in a negative mode to characterize and compare their profiles. These analyses resulted in the detection of 48 compounds (Table 1) distributed in three main categories: (I) phenolic acids (5 compounds); (II) flavones and flavonols (12 compounds); (III) flavan-3-ols (21 compounds). All compounds were characterized using accurate mass information obtained via their pseudo molecular ion [M-H]^−^, fragmentations in MS^2^, and comparison with the available literature. APE contained the largest number of compounds (23 compounds), followed by the OF fraction (19 compounds). The description of the phenolics in APE, AF, OF, and HAPE is presented in Figure 1.

##### Phenolic Acids

Phenolic acids (PA) are commonly present in avocado peel in the form of aglycone and esters of quinic acid [29]. In this study, a hydroxycinnamic acid derivative (compound **1**) was found only in APE. Protocatechuic acid was characterized only in the HAPE, while 5-O-caffeoylquinic acid was detected in the AF and OF. Compound **3** was detected only in OF. It showed a pseudoion [M-H]^−^ at *m/z* 381 and a base peak at *m/z* 135 [M-H-202]^−^, which is characteristic for quinoyl moiety. Literature data allowed to characterize this compound as ethyl chlorogenate [27].

##### Flavonols and Flavone

Among the flavonoids identified, quercetin and its derivatives appeared as the major flavonols. In APE, eight glycosylated quercetins (compounds **5–12**) were detected. Of these, only compounds **5**, **6**, and **10** also occurred in AF. The identification was based on the presence of the typical primary fragment ion at *m/z* 300, related to an *O*-glycosidic cleavage, and of the ion fragments at *m/z* 271 and 255 due to losses of [aglycone-CHO]^−^ and [CO+H_2_O]^−^ [28]. Compound **5**, with a [M-H]^−^ at *m/z* 625, showed the characteristic ions at *m/z* 300, 271, 243, and 151, which correspond to quercetin aglycone. The compound could be identified as quercetin diglucoside due to the neutral loss of two glucose molecules [M-H-325]^−^, compared to the fragmentation pattern reported in the literature [29]. Compound **6** had an [M-H]^−^ at *m/z* 595 and produced MS^2^ fragment ions at *m/z* 300, which is equivalent to the partial loss of a glycosyl and pentosyl residue [M-H-295]^−^. It was identified as quercetin-3-O-arabinoglucoside by comparison with the literature [30]. Compounds **7** and **9** appeared with the same pseudo molecular ion at *m/z* 463 and base peak at *m/z* 300 [M-H-163]^−^, indicating that the quercetin molecule underwent the neutral loss of a hexose group. The two compounds were identified as the isomers quercetin 3-*O*-galactoside, and quercetin 3-*O*-glucoside commonly found co-existing [41]. Compound **8** (*m/z* 477) showed loss of a glucuronide moiety [M-H-177]^−^ and generation of MS^2^ fragment ions at *m/z* 300. It was tentatively identified as quercetin 3-*O*-glucuronide by comparing the pattern of fragmentation reported in the literature [46]. Compound **10** (*m/z* 609) showed the loss of a rutinoside [M-H-309]^−^, resulting in secondary ions at *m/z* 300, 271, and 255. Compared with the literature, this compound could be characterized as quercetin-3-*O*-rutinoside (rutin) [29]. Compounds **11** (*m/z* 579), **12** (*m/z* 561), and **16** (*m/z* 477) were identified as derivatives of quercetin, according to the mass spectrum with a specific fragment at *m/z* 301, 300, 271, 255, and 155. Compound **11** showed a loss of 279 amu, which is equivalent to the elimination of deoxyhexosyl and pentosyl residues [M-H-279]^−^. It was tentatively characterized as quercetin-xylosyl-rhamnoside, based on previously published data of avocado peel compounds [32]. Compounds **12** and **16**, present only in AF, could not be characterized due to the lack of reference substances in the literature.

A glycosylated derivative of kaempferol was detected only in APE. It presented a pseudomolecular ion at *m/z* 593 and formed a fragment typical of the kaempferol aglycone (*m/z* 285), with loss corresponding to a portion of deoxyhexosyl and pentosyl [M-H-308]^−^. The attempt to characterize it as kaempferol *O*-rhamnosyl-glucoside was made by comparison with a previous study of the avocado peel [29].

Only flavonoids in the aglycone form were detected in HAPE and OF. Compound **14**, with an [M-H]^−^ at *m/z* 301, was identified as quercetin due to its characteristic fragment ions at *m/z* 271, 255, and 151 [28]. Compound **15** (*m/z* 285) was tentatively characterized as kaempferol for showing fragment ions at *m/z* 255 and 227, as reported in the literature [34].

##### Flavan-3-Ols

From the flavan-3-ols group, monomers, dimers, and trimers built from flavan-3-ols (epi)catechin were found. Among PACs, a series of dimeric and trimeric procyanidin B-type isomers were identified in APE (compounds **18–20, 23**) and its OF fraction (compounds **17–21, 28, 29, 31**). Only one dimer was detected in AF (compounds **21**). The dimers (compounds **17–21, 29, 31**) showed pseudomolecular ions [M-H]^−^ at *m/z* 577 and primary product ions at *m/z* 425 [M-H-152]^−^, 407 [M-H-170]^−^, 289 [M-H-290]^−^, 245, 125 and 109, consistent with what has been reported for these compounds [6,11]. Among these fragments, *m/z* 289 and *m/z* 425 corresponded to the loss of an (epi)catechin unit and a retro-Diels–Alder (RDA) fission, respectively [29,38]. For the two trimers (compounds **23, 28**), with pseudoions at *m/z* 865, the product ion at *m/z* 577 was also observed, relative to the deprotonated dimer [38].

Compounds **36** (APE) and **32–34, 37** (OF) showed the same fragmentation pattern, with an [M-H]^−^ at *m/z* 451. They mainly generated MS^2^ fragment ions at *m/z* 341 [M-H-110]^−^, due to the neutral loss of one catechol moiety, 189 [M-H-262]^−^ and 109 [M-H-342]^−^. The five compounds were identified as isomers of flavalignan cinchonain I [47]. A probable derivative of cinchonain I was still observed in APE. This compound (**36**), with an [M-H]^−^ at *m/z* 565, showed fragment ions typically reported in the literature for cinchonain I (*m/z* 451 [M-H-114]^−^, 341 and 189). Due to the lack of literature data it was not possible to identify this compound.

Two isomers of the chalcan-flavan-3-ol dimer, with a pseudoion [M-H]^−^ at *m/z* 579, were also detected only in APE (compound **22**) and OF (compound **24**). For these compounds, the major product ion was *m/z* 289 [M-H-290]^−^, corresponding to the deprotonated (epi)catechin. The observed product ions and mass accuracy were consistent with what has been described in the literature for these components [37].

Compound **26** (*m/z* 289), found only in the sample of AF, gave MS^2^ base fragment at *m/z* 109 [M-H-180]^−^ (cleavage of the B or C ring of (epi)catechin), and other fragments consistent with (epi)catechin structure (*m/z* 245, 203, 161, 123). With the information on the fragments of the mass spectrum and the accuracy of the measured mass, it was possible to identify the compound as (epi)catechin.

#### 2.1.2. TPC, PAC and Antioxidant Activity

In Table 2 are shown the total phenolic (TPC) and proanthocyanidin (PAC) content and the antioxidant activity assessed by DPPH free radical scavenging capacity (DPPH), ferric reducing antioxidant power (FRAP), and oxygen radical absorbing capacity (ORAC). The TPC and PAC in the extracts ranged from 244.45 to 297.42 mg gallic acid equivalent (GAE)/g dry extract (DE) and 456.13 to 4708.39 mg catechin equivalent (CaE)/g DE, respectively. The highest TPC was found in APE, followed by OF, HAPE, and AF, whereas for PAC, OF showed the highest values, followed by APE and AF, and no PAC was detected in HAPE. For antioxidant activity, significant differences (*p* ˂ 0.05) in DPPH, FRAP, and ORAC values were found among the extracts. The APE extract had the highest DPPH values (900.4 µM Trolox equivalent antioxidant capacity (TEAC)/g DE), followed sequentially by the OF (706.4 µM TEAC/g DE), HAPE (596.5 µM TEAC/g DE), and AF (502.8 µM TEAC/g DE). Both FRAP and ORAC assays had similar behavior in the antioxidant activity of the extracts; OF present the highest antioxidant capacity (7176.5 µM TEAC/g DE and 15584.6 µM TEAC/g DE, respectively), whereas AF showed the lowest antioxidant potential with values of 2160.6 and 6252.3 µM TEAC/g DE for FRAP and ORAC assays, respectively.

### 2.2. Antibacterial Activity

**Figure 2:**
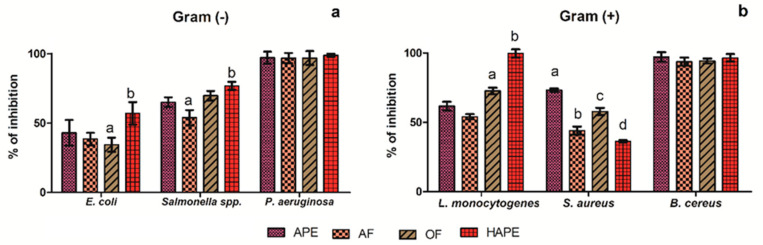
Screening Inhibitory Activity (%) of avocado peel extract and its fractions at 500 µg/mL. a—Gram-negative (−) bacteria: *Escherichia coli (E. coli)*; *Salmonella* spp.; *Pseudomona aeruginosa* (*P. aeruginosa*); and b—Gram-positive (+): *Listeria monocytogenes* (*L. monocytogenes*); *Staphylococcus aureus* (*S. aureus*); *Bacillus cereus* (*B. cereus*). Avocado peel extract (APE); organic fraction (OF); aqueous fraction (AF); acid-microwave hydrolyzed avocado peel extract (HAPE). Different letters on the bars between each bacteria strain represent significant differences according to the Tukey test (*p* < 0.05).

To screen the inhibitory capacity of the APE, OF, AF, and HAPE against Gram (-) and (+) bacteria was assessed by the microdilution test. Overall, *P. aeruginosa* and *B. cereus* were sensible (inhibition ˃95%) to all the extracts at the tested concentration (500 µg/mL) (Figure 2a,b). While the APE moderate inhibited *E. coli* and *Salmonella* spp. (Figure 2a) (43.06 ± 0.19% and 65.02 ± 3.51%, respectively), *E. coli* was less inhibited by the AF and OF (*p* ˂ 0.05), though the inhibition by those fractions was above the 50% for *Salmonella* spp. No significant differences were observed for APE or OF in the percentage of inhibition against *Salmonella* spp. Contrariwise, HAPE actively inhibited the *E. coli* and *Salmonella* spp. (*p* ˂ 0.05) (57.03 ± 8.15% and 76.89 ± 2.86%, respectively).

Alike, Gram (+) (Figure 2b) *L. monocytogenes* showed *(p* ˂ 0.05) sensitivity to OF (72.60 ± 2.34%) and high sensitivity to HAPE (99.65 ± 2.96%). The last results are inverse to the effect of that fraction on *S. aureus* inhibition, in which HAPE showed a low inhibitory effect (*p* ˂ 0.05) (36.32 ± 0.75%), compared to the APE (73.35 ± 1.12%). In this sense, the results indicated that the HAPE, rich in low molecular phenolic compounds, inhibited the bacillary forms of bacteria to a greater extent than the coccoid forms.

In Table 3, the MIC (no microbial growth visually observed) for Gram (-) and (+) bacteria are compared for all the extracts and positive control (amoxicillin). There can be seen that almost all the tested bacteria except *P. aeruginosa* and *B. cereus* were less sensitive to the aqueous fraction (MIC ˃ 1000 µg/mL). Contrarywise, the OF increased up to 25% the inhibition of *Salmonella* spp. (MIC from APE 1000 to 750 µg/mL of OF). Similarly, the results indicated that the hydrolyzation and fractionation with ethyl acetate of APE increased the inhibitory efficiency up to 25% for *E. coli* and *Salmonella* spp., 83.34% for *L. monocytogenes*, compared to the APE. Therefore, the high sensitivity of the last bacteria to HAPE can analogically inhibit and disrupt the preformed biofilm.

The minimal inhibitory concentration (MIC) is expressed as µg/mL of the avocado peel extract (APE), aqueous fraction (AF), organic fraction (OF), and the acid-microwave hydrolyzed avocado peel fraction (HAPE), and amoxicillin as the positive control. Gram-negative (-) bacteria: *Escherichia coli (E. coli); Salmonella* spp.; *Pseudomonas aeruginosa (P. aeruginosa)*; and Gram-positive (+): *Listeria monocytogenes (L. monocytogenes); Staphylococcus aureus (S. aureus); Bacillus cereus (B. cereus).*

### 2.3. Listeria Monocytogenes Biofilm Inhibition and Disruption

**Figure 3:**
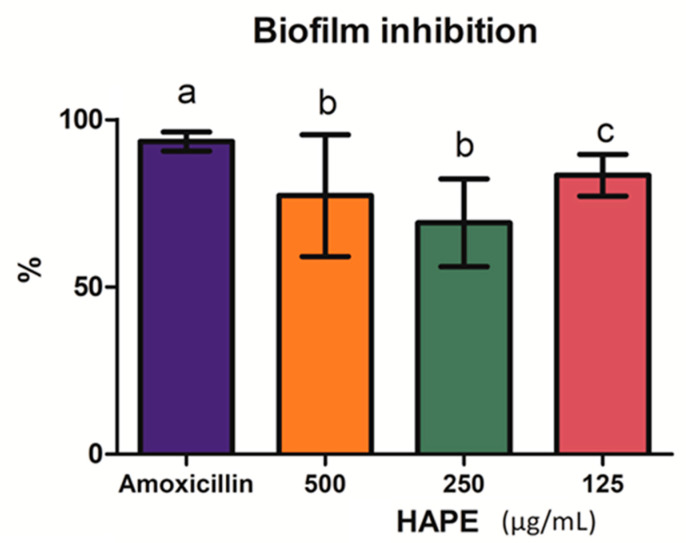
The % of *Listeria monocytogenes* biofilm inhibition by acid-microwave hydrolyzed avocado peel extract (HAPE) at the different concentrations (500, 250, and 125 µg/mL) and amoxicillin (positive control) after 12 h of incubation at 37 °C. Different letters on the bars represent significant differences according to Levene’s test (*p* < 0.05).

The biofilm formation by the *L. monocytogenes* was inhibited above 50% at all treated concentrations (Figure 3). No differences were observed between the inhibition at the upper and middle concentrations (*p* ˃ 0.05) (69.14 ± 13.07% and 77.30 ± 18.18%, respectively), which were (*p* ˂ 0.05) lesser than the lower tested concentration (83.41 ± 6.23%), though inferior to amoxicillin (*p* ˂ 0.05).

**Figure 4:**
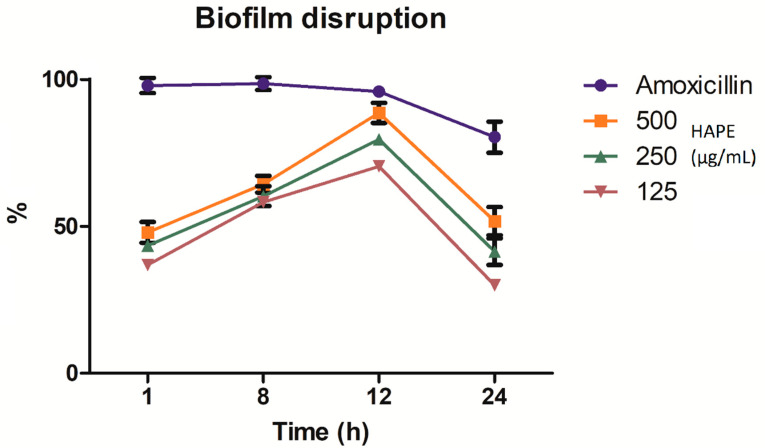
Time-concentration *Listeria monocytogenes* biofilm disruption by acid-microwave hydrolyzed avocado peel extract (HAPE) at different intervals of time (1, 8, 12, and 24 h) at different concentrations (500, 250, and 125 µg/mL) and amoxicillin (positive control) incubated at 37 °C.

Linked to the effect of HAPE on the preformed biofilm on different intervals of time (1, 8, 12, and 24 h), the tested concentrations (500, 250, and 125 µg/mL) presented time-concentration-dependent inhibition (Figure 4). The two-way ANOVA indicated that both concentration-time and their interaction effect are extremely significant *(p* ˂ 0.0001) to the disruption of the preformed biofilm. At short times (1 h), all the concentrations inhibited the biofilm around 50%, without significant differences between the tested concentrations. The trend continued up to 8 h with a slightly inhibitory increase (*p* ˃ 0.05). At 12 h and concentration-dependent, the higher % of the preformed biofilm was reached. A concentration of 500µg/mL did not show significant differences than the positive control (amoxicillin) (Bonferroni’s test *p* ˂ 0.05). However, with the increase in time the % of disruption decreased below 50% in a concentration-dependent way (*p* ˂ 0.05).

## 3. Discussion

Avocado peel is considered to be an excellent source of phenolic compounds to substitute for chemical antioxidants and antimicrobials [3,32]. The raw APE and further fractionation and hydrolysis process led to a change in the TPC, PAC, and antioxidant activity (Table 2). The differences can be attributed to the phenolic composition of the extracts APE, AF, OF, and HAPE (Table 1). While APE and OF exhibited higher individual PC, TPC, and PAC, and more significant antioxidant activity, the AF and HAPE presented the lowest. These variations could be due to the compounds’ polarity and the effect of the hydrolyzation on the APE. Raw extracts, due to the wide composition (PCs, sugars, proteins, and others), have displayed high antioxidant activity [11,48]; nevertheless, some studies have suggested that the weak polar fractions such as those from ethyl acetate display superior antioxidant activity to the aqueous and ethanolic fractions [22,49]. This was attributed to the concentration of molecules with a superior number of hydroxyl groups, such as PAC, flavonoids, and PA with radical, scavenging, reducing, quenching, and metal chelating capacity [50]. There was reported that phenolic compounds associated with flavan-3-ols, flavonols, and hydroxycinnamic derivatives had the highest antioxidant potential [22,51,52]. The antioxidant activity of AF and HAPE was significantly different from that of APE, with a decrease between 40% and 60%, approximately, for the three assays. These results could be due to PCs’ lower content in those fractions, as shown in Table 1. In this sense, acid hydrolyzation catalyzed by temperature decreased the antioxidant activity in phenolic extracts from wheat bran [14]. Also, high temperatures promote the degradation of PCs from plant extracts [53]. Since the antioxidant activity of PCs is driven by the fact that the intramolecular hydrogen bonds allow the stabilization of catechol groups or the regeneration of these catechol groups, with a simultaneous interaction of two catechol radicals to form the quinone; the ABA seems to be related to the affinity toward the lipophilic character of the bacterial membrane, and the modification of the bacterial growth environment [7,54].

Consequently, related to PCs’ nature, they can act at the membrane and non-membrane level. Polyphenols interact with the cell membrane, permeabilizing it and causing its disruption [9]. As a result of the disorder in the cell membrane (e.g., modification of protein function and alteration of lipid order), variations occur in the interchange of nutrients, protons, enzymatic activity, synthesis of proteins and nucleic acids, which ultimately ends up promoting bacteriostatic or bactericidal effects [55,56]. Interestingly, PA can diffuse across the membrane and subsequently dissociate, generating acidification of the cytoplasm affecting the sodium–potassium pump. This effect decreases the proton motive force that drives ATP synthesis, limiting the cell growth and eventually causing its death [15,57]. Apart from the preceding, flavonoids own the ability to inhibit the synthesis of DNA gyrase necessary for genetic material replication [58], and to complex with the lipoproteins affecting the membrane and cell wall functions, inducing the cellular lysis [59]. Besides, PACs, which are polymers of flavan-3-ols, compete for essential micronutrients (e.g., chelation of iron, zinc, and others), inhibit extracellular enzymes, and cause aggregation of the cells, limiting the bacteria growth [2,15,16,17,18].

The structure and polarity of the cell wall and cytoplasmic membrane that characterize Gram (+) bacteria make them more prone than Gram (−) to be damaged by PCs. This is because the latter bacteria are protected with an external lipopolysaccharide membrane that restricts the diffusion of PCs to the target sites [9,19,20]. Thus, certain hydrophilic PCs are excluded. On the other hand, Gram (+) bacteria do not have an external membrane, and therefore the PCs of higher polarity can interact better with the peptidoglycan wall (teichoic and lipoteichoic acids). Additionally, there was observed that PA own a greater ABA than flavonoids, though, in general, the ABA seems to be strain-dependent [59,60,61].

The % of inhibition against Gram (−) and Gram (+) bacteria exhibited by APE, AF, OF, and HAPE was significant. Thus, the inhibitory effects were reflected in MIC values that ranged between ≥125 and ≤1000 μg/mL (Table 3). Usually, MIC above 1000 μg/mL is not considered relevant ABA since a large concentration of the bioactive will be required for clinical applications [16]. The % of inhibition presented for each fraction is related to the phenolic composition with recognized ABA [16,62,63,64,65].

In this sense, raw plant extracts with high phenolic content were shown to inhibit efficiently Gram (−) and (+) bacteria [2,21,22,66]. For instance, raw extract from *Punica granatum* peel showed bacteriostatic and bactericidal effects over *S. aureus*, *P. aeruginosa,* and *B. cereus*. However, a weak inhibition of *Salmonella typhi* and *E. coli* was also displayed [8]. Alike, *L. monocytogenes* exhibited higher sensitivity against grape pomace extract than *S. aureus,* while *E. coli* and *S. typhimurium* presented lower sensitivity [67]. While other plant extracts presented weak inhibition of *B. subtilis, S. aureus* [68], and *E. coli* [69]. Related to APE, employing the same microdilution test, similar MICs were reported (104.2–416.7 μg/mL) for Gram (+) and (−) bacteria [17,70].

Associated to PCs’ polarity, there was observed that ethyl acetate extract from *Galla chinensis* had greater antibacterial and antioxidant activity than ethanolic and aqueous extracts [22]. The authors indicated that almost 60% of PACs presented in such extract were weak polar and high molecular weight tannins, compared to water and ethanolic fractions; thus, they displayed a greater biological activity due to the availability of the pyrogallol group. Similarly, other ethyl acetate fractions high in phenolic acids from various wines exhibited greater inhibitory activity on *E. coli* and *S aureus* compared to the aqueous fractions [69]. Likewise, the procyanidin high ethyl acetate fraction from laurel wood actively inhibited foodborne bacteria (*L. monocytogenes, E. coli, S. enterica, S. aureus, E. faecalis,* and *C. albicans*) with MICs from 500 to 1000 µg/mL. Linked to APE, the high weight PAC fraction of APE limited the adhesion mechanism of *Helicobacter pylori*, inducing its inhibition, while low molecular weight phenolics were less effective [24]. The latter statement also was supported previously by Meyer and colleagues [58], who reported that PACs from grape seed actively inhibited Gram (−) and (+) bacteria, compared to its monomeric flavonoid fractions (catechin and epicatechin).

Individual PCs present in APE, AF, OF, and HAPE owned ABA. Chlorogenic acid and quinic acid displayed a bacteriostatic effect against *E. coli* [64]. These authors indicated that chlorogenic acid inserts the microbial toxicity synergically with other phenolic compounds. Protocatechuic acid in plant extracts was observed to cause the membrane lysis of bacteria [71]. 5-*O*-caffeoylquinic acid from its side presented MICs ranging from 5000 to 10,000 µg/mL against *E. coli, S. aureus, P. aeruginosa*, and other Gram (−) and (+) bacteria [72]. Pernin, Guillier and Dubois-Brissonnet, (2019) [73] indicated that the undissociated forms of PA own better inhibition against *L. monocytogenes,* e.g., quinic acid and caffeic acid that constitute the chlorogenic acid conformation.

Aglycones of flavonoids showed a higher ABA. This was supported by the idea of glycosylation increasing the hydrophilic character or PCs decreasing the interaction with the bacterial membrane [11]. In this sense, quercetin 3-*O*-β-D-Glucoside and rutin showed no inhibition against *E. coli* and *S. aureus,* while the aglycones displayed an MIC of 2000 and 25,000 µg/mL, respectively [4]. Similar results were reported elsewhere [62]; quercetin displayed a moderated inhibition against *E. coli* (MIC ˃ 1000 µg/mL); contrarywise, the quercetin glycoside (rutin) actively inhibited *E. coli, P. aeruginosa*, and *S. aureus* (MIC 500–1000 µg/mL). Alike, *E. coli* was inhibited by rutin and epicatechin (MIC 20 µg/mL), while, *Salmonella parathyphi* was most sensitive to epicatechin than rutin (MIC 15 and 20 µg/mL, respectively) [65]. However, the literature is not conclusive, and the inhibition seems to be more strain-dependent, as it was mentioned above.

Besides high antioxidant activity due to the hydroxylation pattern [49], flavalignan species are recognized to own ABA. Chinchonains present in the ethyl acetate fraction of rhizomes of *Smilax glabra* inhibited *S. aureus* (MIC 200 µg/mL) [74]. Similarly, the preceding bacteria and *E. coli* were inhibited by a chinchonain/quercetin-rich extract from *Secondatia floribunda* (MIC 128 and 64 µg/mL, respectively) [16]. Equivalent, an ethyl acetate fraction from the bark of *Trichilia catigua,* containing a mixture of cinchonains Ia and Ib, inhibited *B*. *cereus, S. aureus, E. coli,* and *P. aeruginosa* better than the aqueous fraction (MIC from 310 to 620 µg/mL) [74].

Related to iridoids, a few studies assessed the ABA of penstemide. Zajdel et al. (2013) [75] reported MICs of the purified compound on *S. aureus* (1180 µg/mL), *P. aeruginosa* (1580 µg/mL), and *E. coli* (2000 µg/mL), though the raw methanolic extract of the penstemon species displayed lower MICs. Seemingly, synergy with other iridoid glucosides, phenylpropanoid glycosides, and other compounds conferred the ABA in those raw extracts.

Comparing the APE with the fractions obtained by its acidification (OF and AF), the presence of different isomers of procyanidin type-B and cinchonain I was detected in OF and the occurrence of (epi)catechin detected in AF. As seen in previous works [76,77], this suggests that the interflavonoid bond of these compounds may have been cleaved, forming carbocation capable of reacting with monomeric (epi)catechin to form stable dimers, even without increasing the temperature.

Subsequently, it was possible to explain the ABA of the APE, AF, and OF. Overall, the APE and OF displayed better ABA than the AF, due to, in the first, a wide range of PCs were presented and synergically conferred the ABA. Simultaneously, the second contained almost all the aglycones and weak polar compounds with high ABA. On the other hand, mostly all the glycosides and some aglycone PCs were present in the AF, limiting the ABA.

Unlike the rest of the tested extracts, protocatechuic acid, quercetin, and quinic acid were the PCs identified in HAPE; thus, the synergistic effect of those PCs conferred the ABA and the anti-biofilm capacity (Figure 1, Figure 2 and Figure 3). In particular, *L. monocytogenes* is a concern, especially in the food industry, due to the capacity to form a resistant biofilm [1]. For its formation, *L. monocytogenes,* first, from the planktonic state, gets reversibly attached to the surface, then, the subpopulation of the bacteria irreversibly remains on the surface tightly adhered to by the extracellular excreted polymers (e.g., proteins and polysaccharides); then, microcolonies form and further configure a multilayer biofilm [1,23,78,79]. Therefore, blocking the initial reversive steps is crucial to avoid biofilm formation [80]. PCs own the ability to inhibit those mechanisms and disrupt the preformed biofilm. In this sense, quercetin, a flavonol with known ABA [9], produced the inhibition of *L. monocytogenes* biofilm formation [78]. These authors indicated that at lower concentrations (60 µg/mL), the main mechanisms of inhibition were focused on reducing the reversible attachment, the amount of the extracellular proteins, and the organization of the bacterial colonies. Similarly, a grape extract high in PA, quercetin, and catechin was observed to inhibit the *L. monocytogenes* cell adherence to stainless surfaces by the bacterial motility and energy surface reduction [79]. Alike, synergic interaction between protocatechuic–gallic acid and quinic–caffeic acids increased the *L. monocytogenes* inhibition [73,79]. Linked to the concentration, some studies reported that at low concentrations of the PC, the *L. monocytogenes* biofilm reduction is statistically similar or significantly above that at higher PC concentrations [23,79,80]. However, concentration dependence was also observed [53,79].

PCs disrupt the preformed biofilm in a concentration- and time-dependent way. In a previous work, Vásquez-Armenta and colleagues [78] reported that quercetin actively inhibited the mature *L. monocytogenes* preformed biofilm without significant differences between the tested concentrations after 1 h of its application [78]. Albeit, similar to our results, a complete inactivation was not observed. This can be explained by the fact that disruption leads to the accumulation of biomass from the damaged outer bacteria that protect the inner living cells; consequently, it could promote further recontamination [78,81]. Comparable results were observed on biofilm disruption and concentration dependency up to 24 h of exposition by procyanidins isolated from laurel wood [23] and grape extracts [82]. The results obtained in the present study strongly agreed with the previous works. The application of HAPE (Figure 2) inhibited the biofilm in a non-concentration-dependent fashion. In contrast, we observed that the disruption of the preformed biofilm (Figure 3) was concentration-dependent, although without significant differences at the earliest incubation times. Such effect upon biofilm integrity became significant at 12 h of incubation. The sharp decrease at 24 h on the % biofilm disruption indicated the ability of *L. monocytogenes* to recover and recontaminate.

Meanwhile, the accompanying acid hydrolysis experiment assisted by microwave heating generated the O-C cleavage of all the glycosylated derivatives of quercetin, proven by the presence of only the quercetin aglycone in HAPE. This method probably also led to the extensive degradation of cinchonain I and B-type procyanidin oligomers, since neither they nor their monomers were detected in HAPE. Other authors reported the hydrolyzation (HCl 2 N and 30 min in boiling water) of red raspberry juice rendered principally protocatechuic and quercetin aglycones, though some catechin, epicatechin, and hydroxycinnamic acids were also detected in low concentrations [51]. Also, the microwave catalyzed hydrolyzation process could lead to the formation of other polymeric compounds with ABA, which could not be identified. Regarding this latter, a possible explanation was observed in extracts from non-roasted and roasted coffee [83]. The results indicated that, despite the ABA attributable to the caffeic, chlorogenic, protocatechuic acids and caffeine present in the coffee extracts, the raw extract from non-roasted coffee did not show any significant ABA. The authors concluded that other substances derived from Maillard reactions (also observed in microwave-assisted extraction [84]) were formed along the thermic process, and could influence the ABA as well.

In conclusion, APE is a valuable source of phenolic compounds with antioxidant and antibacterial activity. The fractionation with weak polar solvents increases its biological activity. For the first time, microwave-assisted acid hydrolysis was performed for APE, resulting in potentiation of the bacterial inhibition, especially against *L. monocytogenes* in planktonic and biofilm forms; nevertheless, the antioxidant activity was reduced as many of the PC were degraded. However, the mechanism by which these conditions resulted in the degradation of these compounds by different heating conditions, and the repercussion on the biological activity remains to be dilucidated, thus, such investigation is being performed by our team. The present study provides essential information about the modifications of plant and agro-waste extracts to obtain high effective antimicrobials.

## 4. Materials and Methods

### 4.1. Chemical Reagents

Ethanol, acetonitrile, methanol, HPLC water, dimethyl sulfoxide (DMSO), hydrochloric acid, acetic acids, formic acid, sodium phosphate, 6-hydroxy-2,5,7,8-tetramethylchroman-2-carboxylic acid (Trolox), sodium hydroxide, sodium sulfate, catechin, gallic acid, trypticase soy broth (TSB), 4-(Dimethylamino)-cinnamaldehyde solution (DMAC), and Folin–Ciocalteu reagent were purchased from Merck (Darmstadt, Germany). Crystal violet, 2,2-Diphenyl-1-picrylhydrazyl (DPPH), 2,4,6-tripyridyl-s-triazine (TPTZ), 2,2′-Azo-bis(2-amidinopropane) dihydrochloride (AAPH), and fluorescein were provided by Sigma-Aldrich Co. (St. Louis, MO, USA).

### 4.2. Avocado Peel Extract Obtainment, Fractionation, and Hydrolysis

The peels from avocado Hass (*Persea americana* Mill) were obtained from small-sized avocado fruits from the north part of Chile, according to a previous characterization [85]. Then, an hydroethanolic avocado crude extract was obtained by the optimal condition of the ultrasound–microwave combined method developed in our previous work [3]. There were mixed 1 g of the dry avocado peel with 25 mL of solvent (80% ethanol and distilled water). Consequently, this was sonicated (15 min) and microwave-irradiated (95.1 s) (ultrasonic-microwave cooperative extractor apparatus CW-2000, Shanghai Xtrust Analytical Instrument, Shanghai, China). The extracts were then filtered, the solvent was vacuum removed, and the extract was lyophilized. The APE was kept at −20 °C until use.

#### 4.2.1. Organic and Aqueous Fraction Obtainment

A total of 5 g of APE was diluted in acidified water (HCl 5%, pH 2–3). Then, liquid–liquid extraction was performed with ethyl acetate. The operation was repeated several times until the organic solvent showed no further coloration. The organic fraction (OF) was treated with sodium sulfate and filtered (paper filter Whatman 1). The aqueous fraction was filtered as well. Both fractions were kept separately at −80 °C until drying.

#### 4.2.2. Microwave-assisted Hydrolysis:

A total of 5 g of APE was diluted in 2 M HCl containing 1% ascorbic acid; immediately, it was microwave-irradiated (500 W, 2400 MHz) for 120 sec with resting intervals of 10 min (ultrasonic-microwave cooperative extractor apparatus CW-2000, Shanghai Xtrust Analytical Instrument, Shanghai, China). The temperature was set to not to surpass 70 °C. The cycle was repeated six times. Once the hydrolysis was finished, the extract was cooled down, and the pH was stabilized (pH 3–4) with sodium hydroxide (1% *w*/*v*). The fractioning was performed by following the abovementioned procedure with ethyl acetate. The aqueous fraction was discarded.

For the avocado peel extract (APE), the aqueous fraction (AF), the organic fraction (OF), and the acidic-microwave hydrolyzed organic fraction of APE (HAPE), the solvent was removed separately in a vacuum evaporator, Laborota 4000 (Heidolph, Germany) at 40 °C from 240 to 70 mbar. Then, all the extracts were lyophilized separately and kept at −80 °C until analysis.

### 4.3. Phytochemical Analysis:

Stocks of diluted methanol extracts (10 mg/mL) were used for phytochemical characterization. The Folin–Ciocalteu method was employed to measure TPC as previously published [3]. Briefly, 150 µL of diluted (1:10) Folin–Ciocalteu reagent was mixed with 30 µL of the diluted methanol extract (1:40), and 120 µL of sodium carbonate (7.5%) were mixed and allowed to stand to react for 40 min. The absorbance was recorded at 760 nm, the TPC was expressed in mg gallic acid equivalent (GAE) per gram of dry extract (DE) (mg GAE/g DE). PAC was measured using DMAC reagent (0.6 g, 13 mL of HCl 37% and 187 mL of ethanol); 70 µL of the diluted sample was reacted with 230 µL of the previous reagent for 20 min, and then absorbances were measured at 640 nm. The results were expressed in mg catechin equivalent (CaE/g DE).

#### Chromatographical Analysis

The extracts were analyzed using an HPLC-qTOF-MS/MS on a maXis G3 quadrupole time-of-flight mass spectrometer (Bruker Daltonics, Bremen, Germany) equipped with an electrospray (ESI) source. The MS was connected to a Shimadzu CBM-20A system with communication module CBM-20A, diode-array detector SPD-20A, and a Phenomenex Luna C-18 reversed-phase column (250 × 4.6 mm, five μm) (Torrance, CA, USA).

The chromatographic separation was performed at a flow rate of 1 mL/min and an oven temperature of 40 °C as was stated elsewhere [86]. The mobile phase solvents were aqueous 0.1% formic acid (solvent A) and acetonitrile (solvent B). The gradient program used for the separation was 0 min, 2% B; 0–60 min, 2–100% B. The injection volume was 10 µL, and the run was monitored at 280 nm.

The metabolites were ionized with the negative mode (ESI^−^) with a data acquisition range of 2 scans/s. The mass spectra were scanned from 50–1500 *m/z*. The eluent flow of 200 µL/min was carried to the ion source. Dry N_2_ was used as a nebulizer gas, with a pressure of 2 bar, corresponding to a gas volume flow of 8 L/min. Other ESI conditions were: capillary tension of 4500 V, endplate offset of 500 V, drying temperature of 200 °C, collision energy of 6.0 eV.

### 4.4. Antioxidant Assays

The antioxidant activity of the fractions was assessed employing the methodologies developed elsewhere with modifications [87]. All the activities were expressed as micromoles of Trolox equivalent per gram of dry extract. The curves linearly ranged from 25 to 1000 µM TEAC, but 12.5 to 50 µM TEAC for ORAC.

#### 4.4.1. DPPH Scavenging Capacity

To measure the ability of the bioactive to scavenge the 2,2-Diphenyl-1-picrylhydrazyl (DPPH) radical, 280 µL, the reagent diluted in methanol (Absorbance 1.1) was mixed with 20 µL of the adequately diluted extract (1:40), allowing to react for 30 min. Absorbances were measured at 517 nm.

#### 4.4.2. FRAP

The ferric reducing antioxidant power (FRAP) was assessed literally according to the previous study cited in this item [87]. Briefly, the following stock solutions were prepared: acetate buffer (300 mM, pH 3.6), 10 mM TPTZ in HCl (40 mM), FeCl_3_ · 6H_2_O (20 mM) previously. On the day of the analysis, the FRAP reagent was prepared, mixing 25 mL acetate buffer, 2.5 mL TPTZ solution, 2.5 mL FeCl_3_ · 6H_2_O, and then heated up (37 °C). Then, 150 µL of the diluted extract (1:40) was immediately mixed with 2850 µL of the FRAP solution and allowed to react for 30 min in the dark condition. An amount of 250 µL of the reaction was poured into a 96-well plate. The absorbances of the colored reaction [ferrous tripyridyltriazine complex] were measured at 593 nm.

#### 4.4.3. ORAC

The neutralizing ability of the fraction against the oxygen-derived radicals measured by ORAC Assay [87] was performed employing plate reader Perkin Elmer Victor X2 (Cridersville, OH, USA). Dilution of the bioactive and the analysis was performed in phosphate buffer pH 7.4. For the reaction: 45 µL of the diluted sample (1:40) was mixed with 175 µL fluorescein (108 nM in well), then 50 µL (18 mM in well) of freshly prepared AAPH as a source or the peroxyl radical were added to 96-well plates with black flat bottom wells. Fluorescence conditions were as follows: 37 °C, excitation at 485 nm and emission at 535 nm, for 5 h.

### 4.5. Antibacterial Bioassay

#### 4.5.1. Culture Media and Microbial Identification

The strains included in this assay were Gram-negative: *Escherichia coli*, *Salmonella* spp., and *Pseudomonas aeruginosa;* Gram-positive: *Listeria monocytogenes*, *Staphylococcus aureus, Bacillus cereus.* The bacteria were provided by Prof. Juan Esteban Reyes (Laboratory of Microbiology, Universidad del Bío-Bío, Chile). Bacteria were maintained in trypticase soy broth (TSB) medium containing 50% (v/v) glycerol at −80 °C.

For the tests, previously, a 100 μL aliquot of each stocked bacterial suspension was transferred to TSB medium and allowed to grow aerobically for 18 h at 37 °C. To reach the appropriate number of colony-forming units (CFU/mL) for each bacterium per test, 10 μL of each inoculum was diluted 100×, and the suspension was counted by Neubauer’s Chamber.

#### 4.5.2. Broth Microdilution Assay (Planktonic)

The antimicrobial test was performed according to our previous work [3], with some modifications. The extract and fractions were dissolved in sterile distilled water with 5% of DMSO at 10 mg/mL (to reach a final in-well concentration of 500 µg/mL), and filtered (0.2 µm sterile filter). Inoculum containing 2–3 × 10^4^ CFU/mL was added to each well. The in vitro assay was added to 190 μL of inoculum and 10 μL of the sample solution in each well. The other wells contained the sample blank (190 μL of TSB and 10 μL sample solution), growth control (200 μL of inoculum), negative control (200 μL TSB), solvent control (190 μL TSB medium and 10 μL solvent). For positive control, amoxicillin dissolved in the sterile broth was employed (10 μL and 190 µL of inoculum). Plates were incubated overnight (12–18 h) at 37 °C in a growth incubator.

For this experiment, two concepts were used to define the antibacterial activity: The % of inhibition spectrophotometrically assessed at 550 nm and the minimal inhibitory concentration (MIC), determined as the lowest concentration that visually inhibited above 95% of the bacterial growth [80].

#### 4.5.3. *L. monocytogenes* Biofilm Inhibition and Disruption

The biofilm inhibition and disruption were assessed referenced to a published work with modifications [23]. The capability of inhibiting the biofilm formation was quantified employing the crystal violet assay as follows:

Following the planktonic test (see the previous item), *L. monocytogenes* inoculum were treated with the most active fraction at the defined concentrations. Positive control (amoxicillin 10 µg/mL), negative controls, control growth, solvent control, and sample blank for each concentration were introduced. Then, after the overnight, the capacity to inhibit the biofilm was assessed.

Accordingly, plates were drained, washed with sterile water 3 times, and dried at 60 °C for 45 min. Then 100 µL of crystal violet (0.5%) was poured into the well and stained for 5 min. Consequently, the dye was removed with distilled water (5 times), plates were dried at the antecedent conditions. Absorbances were recorded at 595 nm.

For disruption measurement, the biofilm was performed employing the *L. monocytogenes* inoculum prepared as per microdilution assay. A total of 100 µL of the inoculum was added to wells for each tested concentration, solvent, growth, and positive controls, respectively. The same volume of TSB was poured for the sample blank and negative control. The plates were incubated at 37 °C for 18 h. Once the biofilm was formed, the plates were drained, washed with sterile distilled water 3 times, and dried (60 °C for 30–40 min). Then, 110 µL of each concentration of the active fraction (diluted in TSB and filtered) was pipetted into each well; amoxicillin, solvent, and broth were added onto the positive, solvent, negative, and growth controls, respectively. Plates were incubated for 1, 8, 12, and 24 h at 37 °C. The remaining biomass was quantified by crystal violet. Plates were washed and stained as described above. After the washing and drying, the plates were chilled down, and 110 µL of ethanol was added to each well. Finally, 100 µL microliters of the biomass were pipetted and poured into a new plate. The absorbances were recorded at 595 nm.

The percent of planktonic and biofilm inhibition and disruption was measured using the following formula:

$$\%inhibition=100-\left(\frac{\left(XS-XB\right)}{XC}\right)*100$$

where: ***XS*** is the absorbance of the sample, ***XB*** is the absorbance of the respective blank, and the ***XC*** is the absorbance of the control growth. Negative control for all the experiments was considered the blank for solvent, positive, and growth controls.

All the absorbances in the present work were measured employing a spectrophotometer microplate reader Epoch 256695 (BioTek, Winooski, VT, USA).

The work scheme followed is represented in Appendix A.

### 4.6. Statistical Analysis

The phytochemical characterization and antioxidant activity are reported as the average of three measurements with the standard deviation. Antimicrobial activity was performed duplicated with at least three measures each, with at least two replicates, repeated in different days.

Differences were addressed by one-way or two-way ANOVA and Tukey’s or Bonferroni post hoc test, depending on the homogeneity of variances. A Kruskal–Wallis test and Levene’s post hoc test were applied for those variables that did not have a normal distribution. A 95% confidence level was applied for all the statistical analyses using STATGRAPHICS Centurion XVI.I.

## Figures and Tables

**Table 1:** High-resolution mass spectrometry (MS) data and negative ion MS^2^ fragmentation of compounds characterized in avocado peel extract (APE), the aqueous fraction (AF), the organic fraction (OF), and the organic fraction of acid-microwave hydrolyzed avocado peel extract (HAPE)

N°	*tR* (min)	Molecular Formula	[M-H]^−^ (*m/z*)	Δ ppm	MS2 Fragments *m/z* (% Base Peak)	Proposed Compound	Sample				Ref.
							APE	AF	OF	HAPE	
***Phenolic acids***											
**1**	10.0	C_32_H_36_O_18_	707.1864	6.51	353 (97), 191 (100)	chlorogenic acid dimer	+	−	−	−	[25]
**2**	10.5	C_7_H_6_O_4_	153.0191	5.65	153 (32), 109 (100)	protocatechuic acid	−	−	−	+	[26]
**3**	16.4	C_18_H_22_O_9_	381.1185	1.29	381 (43), 179 (51), 161 (22), 135 (100)	ethyl chlorogenate	−	−	+	−	[27]
**4**	17.3	C_16_H_18_O_9_	353.0864	−0.87	191 (100)	5-*O*-caffeoylquinic acid	−	+	+	−	[28]
***Flavonols***											
**5**	11.9	C_27_H_30_O_17_	625.1398	−0.20	301 (19), 300 (100), 271 (58), 255 (31), 243 (40), 151 (21)	quercetin diglucoside	+	+	−	−	[29]
**6**	12.7	C_26_H_28_O_16_	595.1285	0.57	301 (22), 300 (100), 271 (55), 255 (35), 243 (21), 151 (12)	quercetin 3-*O*-arabinoglucoside	+	+	−	−	[30]
**7**	14.0	C_21_H_20_O_12_	463.0888	3.66	463 (47), 300 (100), 301 (64), 271 (85)	quercetin 3-*O*-galactoside	+	−	−	−	[31]
**8**	14.1	C_21_H_18_O_13_	477.0686	4.68	477 (11), 301 (100), 255 (6), 179 (5)	quercetin 3-*O*-glucuronide	+	−	−	−	[29]
**9**	14.2	C_21_H_20_O_12_	463.0882	2.37	463 (45), 301 (97), 300 (100), 271 (83)	quercetin 3-*O*-glucoside	+	−	−	−	[31]
**10**	14.5	C_27_H_30_O_16_	609.1448	−0.34	300 (100), 271 (66), 255 (40), 243 (36)	quercetin 3-*O*-rutinoside	+	+	−	−	[29]
**11**	15.2	C_26_H_28_O_15_	579.1316	−4.91	579 (34), 301 (33), 300 (100), 271 (35), 255 (11)	quercetin xylosyl-rhamnoside	+	−	−	−	[32]
**12**	15.6	..	561.0736	..	447 (79), 301 (79), 300 (100), 271 (21), 255 (26)	quercetin derivative	+	−	−	−	[32]
**13**	15.7	C_27_H_30_O_15_	593.1469	5.39	593 (23), 285 (37), 284 (100), 255 (33)	kaempferol *O*-hexosyl-deoxyhexose	+	−	−	−	[33]
**14**	20.6	C_15_H_10_O_7_	301.0343	0.07	301 (31), 178 (34), 151 (100)	Quercetin	−	−	+	+	[29]
**15**	23.4	C_15_H_10_O_6_	285.0400	2.23	285 (100), 151 (10)	Kaempferol	−	−	+	−	[34]
**16**	28.1	C_21_H_18_O_13_	477.0665	2.79	301 (39), 179 (52), 151 (100), 121 (56)	quercetin derivative	−	−	+	−	[32]
***Flavan-3-ols***											
**17**	9.3	C_30_H_26_O_12_	577.1337	−0.61	407 (8), 289 (9), 245 (10), 161 (23), 151 (27), 125 (100), 109 (21)	procyanidin dimer B ^a^	−	−	+	−	[29,35,36]
**18**	9.5	C_30_H_26_O_12_	577.1372	5.45	577 (33), 425(37), 407 (66), 289 (100), 243 (17)	procyanidin dimer B ^a^	+	−	−	−	[29,35,36]
**19**	10.3	C_30_H_26_O_12_	577.1366	4.41	577 (33), 425(60), 407 (52), 289 (100), 245 (16)	procyanidin dimer B ^a^	+	−	−	−	[29,35,36]
**20**	10.6	C_30_H_26_O_12_	577.1352	1.99	577 (58), 451 (9), 425(84), 407 (66), 289 (100), 245 (17)	procyanidin dimer B ^a^	+	−	−	−	[29,35,36]
**21**	11.3	C_30_H_26_O_12_	577.1339	−0.26	407 (12), 289 (14), 245 (17), 161 (30), 151 (21), 125 (100), 109 (39)	procyanidin dimer B ^a^	−	+	+	−	[29,35,36]
**22**	11.5	C_30_H_28_O_12_	579.1512	2.58	289 (100), 245 (11), 203 (6)	chalcan-flavan-3-ol dimer ^b^	+	−	−	−	[37,38]
**23**	11.7	C_45_H_38_O_18_	865.2008	4.0	865 (4), 577 (9), 451 (9), 425 (14), 407 (52), 289 (100), 287 (82), 261 (19), 243 (32)	procyanidin trimer B ^c^	+	−	−	−	[29,35,36]
**24**	11.9	C_30_H_28_O_12_	579.1498	0.01	289 (22), 245 (27), 203 (27), 151 (36), 137 (48), 125 (69), 123 (80), 109 (100)	chalcan-flavan-3-ol dimer ^b^	−	−	+	−	[37,38]
**25**	12.1	C_30_H_26_O_11_	561.1348	−7.73	561 (15), 407 (6), 289 (100), 245 (8)	(epi)afzelechin–(epi)catechin	+	−	−	−	[39]
**26**	12.2	C_21_H_24_O_11_	451.1231	−0.86	289 (8) 245 (23), 123 (83), 109 (100)	catechin-3-*O*-glucoside	−	−	+	−	[35]
**27**	12.3	C_15_H_14_O_6_	289.0712	1.85	289 (23), 245 (18), 203 (32), 161 (42), 123 (80), 109 (100)	(epi)catechin	−	+	−	−	[29,35]
**28**	12.4	C_45_H_38_O_18_	865.1954	−2.35	407 (27), 289 (18), 243 (8), 161 (39), 137 (33), 125(100)	procyanidin trimer B ^c^	−	−	+	−	[29,35,36]
**29**	13.7	C_30_H_26_O_12_	577.1333	−1.30	407 (17), 289 (10), 245 (10), 161 (9), 137 (37), 125 (100), 123 (28), 109 (25)	procyanidin dimer B ^a^	−	−	+	−	[29,35,36]
**30**	14.1	C_39_H_32_O_15_	739.1642	−2.09	289 (21), 245 (12), 177 (100), 161 (31), 137 (34), 125 (35), 109 (60)	procyanidin dimer monoglycoside	−	−	+	−	[40]
**31**	14.4	C_30_H_26_O_12_	577.1343	0.43	407 (11), 289 (13), 245 (10), 161 (35), 137 (32), 125 (100), 123 (24), 109 (26)	procyanidin dimer B ^a^	−	−	+	−	[29,35,36]
**32**	14.4	C_24_H_20_O_9_	451.1024	0.09	341 (13), 217 (57), 189 (85), 177 (34), 161 (35), 133 (35), 123 (33), 109 (100)	cinchonain I ^d^	−	−	+	−	[29,41]
**33**	15.2	C_24_H_20_O_9_	451.1031	1.64	341 (9), 217 (26), 189 (100), 177 (38), 161 (36), 133 (25), 123 (34), 109 (56)	cinchonain I ^d^	−	−	+	−	[29,41]
**34**	15.8	C_24_H_20_O_9_	451.1029	1.20	407 (10), 341 (16), 255 (34), 217 (25), 189 (65), 177 (54), 161 (27), 123 (30), 109 (100)	cinchonain I ^d^	−	−	+	−	[29,41]
**35**	17.5	C_24_H_22_O_16_	565.0834	1.7	451 (100), 341 (87), 217 (40), 189 (34)	cinchonain I derivative ^d^	+	−	−	−	[29,41]
**36**	17.8	C_24_H_20_O_9_	451.0997	−5.89	451 (10), 341 (100), 189 (9)	cinchonain I ^d^	+	−	−	−	[29,41]
**37**	18.2	C_24_H_20_O_9_	451.1030	1.42	341 (13), 217 (40), 189 (100), 177 (41), 161 (34), 151 (17), 133 (27), 123 (33), 109 (85)	cinchonain I ^d^	−	−	+	−	[29,41]
***Other compounds***											
**38**	2.5	..	533.1737	..	533 (2), 191 (100)	quinic acid derivative	+	−	−	−	[42]
**39**	2.5	..	383.1180	..	341 (11), 191 (100)	quinic acid derivative	+	−	−	−	[42]
**40**	2.5	..	305.0483	..	191 (100)	quinic acid derivative	+	−	−	−	[42]
**41**	2.7	..	249.0145	..	249 (3), 211 (14), 191 (100), 171 (11), 127 (18), 101 (10)	quinic acid derivative	−	+	−	−	[42]
**42**	3.0	C_12_H_22_O_11_Cl	377.0853	2.07	377 (45), 341 (100), 215 (20)	sucrose	−	+	−	−	[43]
**43**	7.7	C_14_H_24_O_12_	383.1217	1.56	191 (100)	quinic acid dimer	−	−	+	−	[43]
**44**	9.6	C_7_H_12_O_6_	191.0561	5.02	191 (100), 171 (10), 127 (18), 111 (8), 109 (18)	quinic acid	−	−	−	+	[29]
**45**	10.1	..	467.0790	..	353(49), 191 (100)	quinic acid derivative	+	−	−	−	[29]
**46**	10.5	C_21_H_32_O_10_	443.1919	1.64	443 (19), 119 (51), 113 (40), 101 (100)	penstemide	−	+	−	−	[29]
**47**	11.4	C_18_H_26_O_10_HCOOH	401.1466	2.38	401 (100), 269 (28)	benzyl alcohol hexose pentose	+	−	−	−	[44]
**48**	22.5	C_8_H_12_O_7_	219.0505	2.61	219 (2), 111 (100)	ethyl citrate	−	−	−	−	[45]

A + means that the compound was present in the sample. − means that the compound was not present in the sample. Compounds characterized according to the high-resolution mass spectrometry (HRMS) and MS^2^; *t_R_*: retention time (in minutes); Δ ppm: mass accuracy errors. ^a^ the stereochemistry of the compound is not resolved; thus, this compound could be procyanidin B1–B8. ^b^ the stereochemistry of the compound is not resolved. ^c^ the stereochemistry of the compound is not resolved. ^d^ the stereochemistry of the compound is not resolved; thus, this compound could be cinchonain-Ia, Ib, Ic, or Id.

**Table 2:** Phytochemical characteristics and antioxidant activity of avocado peel fractions.

Sample		TPC	PAC	DPPH	FRAP	ORAC
	Assay	mg GAE/g DE	mg CaE/g DE	µM TEAC/g DE		
APE		297.42 ± 10.7 b	2535.43 ± 65.56 a	900.4 ± 8.8 d	4954.2 ± 100.8 d	12541.2 ± 574.8 d
OF		282.98 ± 12.87 b	4708.39 ± 177.96 c	706.4 ± 29.3 c	7176.5 ± 142.9 c	15584.6 ± 268.1 c
AF		244.45 ± 8.65 a	456.13 ± 9.37 b	502.8 ± 8.9 ba	2160.6 ± 137.7 a	6252.3 ± 267.8 a
HAPE		252.12 ± 10.79 a	ND	596.5 ± 9.1 b	2935.2 ± 124.9 b	8930.3 ± 409.4 b

Means (n = 3) ± standard deviations are reported per gram of dry extract (DE). Different letters in the same column represent significant differences according to the Tukey test (*p* < 0.05). Abbreviations: APE—avocado peel extract; OF—organic fraction; AF–aqueous fraction; HAPE—acid-microwave hydrolyzed avocado peel extract. TPC—total phenolic content, expressed as milligrams of gallic acid equivalent (GAE); PAC—proanthocyanidin content, expressed as milligrams of catechin equivalent (CaE). DPPH—2,2-Diphenyl-1-picrylhydrazyl; FRAP—ferric reducing antioxidant power; ORAC—oxygen radical absorbance capacity; TEAC—Trolox equivalent antioxidant capacity; ND—non-detected.

**Table 3:** Minimal inhibitory concentration (MIC) for avocado peel extract, the fractions, and positive control.

Extract		MIC µg/mL					
	Strain	*E. coli*	*Salmonella* spp.	*P. aeruginosa*	*L. monocytogenes*	*S. aureus*	*B. cereus*
Amoxicillin		≥10	≥1000	≥125	≥10	≥25	≥10
APE		≥1000	≥1000	≥500	≥750	≥750	≥500
AF		≥1000	≥1000	≥500	≥1000	≥1000	≥500
OF		≥1000	≥750	≥500	≥750	≥1000	≥500
HAPE		≥750	≥750	≥500	≥125	≥1000	≥500

**Figure A1:**
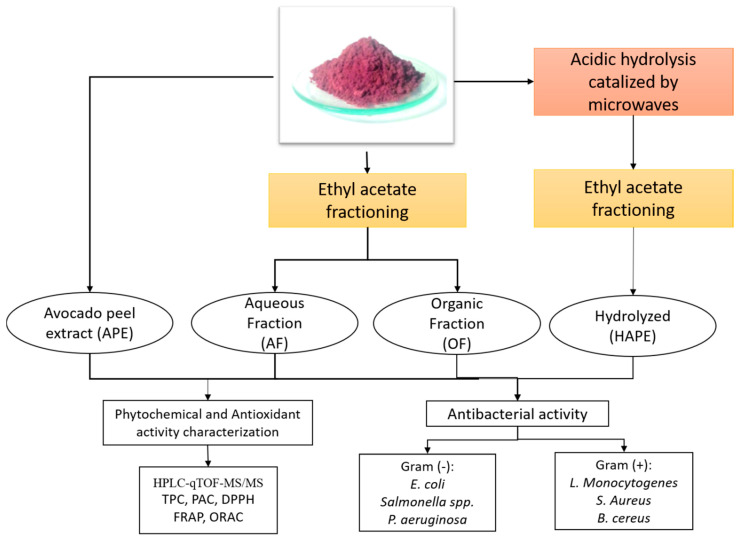
Experimental work chart. Abbreviations: high-performance liquid chromatography–quadrupole time-of-flight mass/mass spectrometry (HPLC-qTOF-MS/MS); Total Phenolic Content (TPC); Proanthocyanidin Content (PAC); 2,2-Diphenyl-1-picrylhydrazyl (DPPH) radical scavenging capacity; ferric reducing antioxidant power (FRAP); oxygen radical absorbance capacity (ORAC); *Escherichia* *coli* (*E. coli*); *Salmonella* spp.; *Pseudomonas aeruginosa (P. aeruginosa); Listeria monocytogenes (L. monocytogenes)*; *Staphylococcus aureus (S. aureus); Bacillus cereus (B. cereus).*
